# Combination therapy of doxorubicin and Sildenafil inhibits the growth of pediatric rhabdomyosarcoma

**DOI:** 10.1007/s00432-022-04092-0

**Published:** 2022-06-28

**Authors:** Cristian Urla, Matias Julian Stagno, Jörg Fuchs, Steven Walter Warmann, Evi Schmid

**Affiliations:** Department of Pediatric Surgery and Pediatric Urology, University Children’s Hospital of Tuebingen, Hoppe-Seyler-Str. 3, 72076 Tübingen, Germany

**Keywords:** Sildenafil, Rhabdomyosarcoma, Apoptosis, Cell proliferation, Clonal cell growth

## Abstract

**Background:**

Overexpression of phosphodiesterase type 5 (PDE5) has been detected in many types of malignant tumors. Sildenafil, a potent and selective inhibitor of a cGMP-specific PDE5, has been found to enhance the cytotoxic activity of different chemotherapeutic agents including doxorubicin. The combined therapy of doxorubicin with Sildenafil might reduce the possible side effects of chemotherapy while maintaining synergistic anticancer activity. The present study explored for the first time the effects of Sildenafil, alone and in combination with doxorubicin, on pediatric RMS cells.

**Methods:**

Human RMS cells lines RH30 and RD were used. Western blotting and RT-PCR were performed to analyze PDE5 expression in RMS cells. Cell viability was determined using MTT assay. Cell migration was analyzed via transwell chambers, clonal growth and mitotic cell death were analyzed using the clonogenic assay. FACS analysis was performed to evaluate reactive oxygen species (ROS) and apoptosis.

**Results:**

Sildenafil significantly decreased cell viability and migration of RD and RH30 cells. The exposure of RMS cells to doxorubicin resulted in a dose-dependent decrease in their viability. Simultaneous administration of Sildenafil enhanced this effect. The incubation of the RMS cells with Sildenafil in the presence of doxorubicin significantly increased the proportion of apoptotic cells and ROS production compared to the treatment with Sildenafil alone.

**Conclusions:**

The results of our study indicate a link between PDE5 inhibition by Sildenafil and decreased calcium signalling leading to significantly impaired viability, migration, and colony forming of RMS cells. Increased ROS production and apoptosis are mechanisms relevantly contributing to this observation.

## Introduction

Rhabdomyosarcoma (RMS) is the most common soft tissue sarcoma in children. About two-thirds of all sarcomas and approximately 8% of all solid malignant tumors in childhood are rhabdomyosarcomas (Seitz et al. 2007). RMS has two major histologic subtypes, embryonal (ERMS) and alveolar (ARMS), each with distinct clinical, molecular, and genetic features (Chen et al. 2013). While there have been improvements in tumor-directed therapy and supportive care, advances in RMS outcomes over the past two decades were rather modest. Treatment results are limited due to recurrent disease, development of metastases, and multi-drug resistance (Seitz et al. 2007; Chen et al. 2013; Zenitani et al. 2016). Therefore, the development of novel therapeutic strategies against this malignancy is of critical importance.

Sildenafil is a potent and selective inhibitor of a cGMP-specific phosphodiesterase type 5 (PDE5), which is commonly used to treat erectile dysfunction or pulmonary arterial hypertension. Because of its structural similarities with cGMP, it competitively binds to PDE5, leading to increased cGMP levels, thereby activating protein kinase G (PKG), resulting in relaxation of vascular smooth muscle and the increase of blood flow (Mei et al. 2015).

PDE5 is widely expressed in the vascular, pulmonary and visceral smooth muscles, platelets, gastrointestinal epithelial cells, and cerebellum (Carson and Lue 2005). However, PDE5 overexpression has also been detected in many types of malignant tumors (Bender and Beavo 2006; Karami-Tehrani et al. 2012; Zhang et al. 2012). These findings suggest that PDE5 may play an important role in tumorigenesis and might be a promising target for anticancer treatment (Mei et al. 2015). Sarfati et al. reported that Sildenafil and Vardenafil suppress tumor cell growth and induce caspase-dependent apoptosis of B-cell chronic lymphocytic leukemia cells in vitro (Sarfati et al. 2003). Investigating the effects of Sildenafil on tumor growth in various cancer cell lines, Chen et al. reported that treatment of colon cancer cells with low concentrations of Sildenafil was associated with decreased proliferation of cancer cells and increased apoptosis in vitro and resulted in impaired tumor growth in vivo (Chen et al. 2020). Additionally, Das et al. observed that combining Sildenafil and doxorubicin enhances the destruction of ovarian cancer and sarcoma cells, suggesting a potential utility of Sildenafil in chemo-sensitization in suitable malignancies (Das et al. 2010).

Sildenafil is currently not approved by the Federal Drug Administration (FDA) for use in the paediatric population, however, it has been extensively used off-label for paediatric pulmonary hypertension since 2005 (Cohen et al. 2019). Thus, Sildenafil may represent an interesting candidate for anticancer therapy in children.

The aim of the present study was to investigate the effects of Sildenafil on tumor growth and functional mechanisms involved in paediatric RMS cell lines. We also investigated possible sensitizing effects of Sildenafil on the doxorubicin treatment of RMS cells.

## Material and methods

### Cell lines and culture conditions

The embryonal RMS cell line RD (ATCC, Manassas, VA, USA), the alveolar RMS cell line RH30 (DSMZ, Braunschweig, Germany), and the human skeletal muscle cells (SkMC; Sigma Aldrich, Taufkirchen, Germany) were routinely cultured in DMEM medium (Biochrom) supplemented with 10% FCS, 1% penicillin/streptomycin, and 1% L-glutamine (all supplements from Biochrom) in a humidified atmosphere containing 5% CO_2_ at 37 °C. Cells were obtained directly from cell bank (ATCC, DSMZ, date of the procurement: 08/2020) that performs cell line characterizations and passaged in our laboratory for fewer than 6 months after receipt. All cells were tested to be mycoplasma negative (Lonza). Cells were treated with the PDE5 inhibitor Sildenafil (Sigma Aldrich; Merck KGaA, diluted in DMSO in 10 mM) in the absence and/or presence of doxorubicin for the indicated periods and with the indicated concentrations.

### Real-time PCR

Determination of PDE5 transcript levels was performed by RT-PCR. Total RNA was extracted from RD and RH30 cells using RNeasy Mini kit (Qiagen) according to the manufacturer’s instructions. Reverse transcription of total RNA was performed using High capacity cDNA Reverse Transcription Kit (Applied Biosystems) according to the manufacturer’s instructions. Polymerase chain reaction (PCR) amplification of the respective genes was set up in a total volume of 20 μl using 40 ng of cDNA, 500 nM forward a reverse primer and 2 × GoTaq^®^ qPCR Master Mix (Promega) according to the manufacturer’s protocol. Cycling conditions were as follows: initial denaturation at 95 °C for 5 min, followed by 40 cycles of 57 °C for 30 s and 72 °C for 20 s. For the amplification the following primers were used (5’-3’orientaion):

PDE5 fw: ACCGCTATTCCCTGTTCCTT.

PDE5 rev: AAGGTCAAGCAGCACCTGAT.

TBP fw: GCC CGA AAC GCC GAA TAT.

TBP rev: CCG TGG TTC GTG GCT CTC.

Specificity of the PCR product was confirmed by melting curve analysis. Real-time PCRs were performed on a CFX96 Real-Time System (Bio-Rad). All four experiments were done in duplicates. Amplification of the house-keeping gene TBP (TATA-binding protein) was performed to standardize the amount of sample RNA. Relative quantification of gene expression was achieved using the ΔCt method.

### Western blotting

To analyze the expression levels of PDE5, whole-cell extracts were prepared using RIPA buffer (Cell Signaling Technology, Inc.) incubated on ice for 30 min. The whole-cell extracts were centrifuged at 4 °C, 14,000×*g* for 20 min, and the protein concentration of the supernatant was determined by Bradford assay (Bio-Rad labraratories GmbH). Lysates (30 µg) were subjected to 10% SDS-PAGE, and proteins transferred to a nitrocellulose membrane (VWR International GmbH). Membranes were blocked for 1 h at room temperature (RT) with 10% non-fat dried milk (Carl Roth GmbH & Co. KG) in TBS (Sigma-Aldrich; Merck KGaA) containing 0.1% Tween-20 (Carl Roth GmbH & Co. KG). For immunoblotting, the membranes were incubated overnight at 4 °C with an antibody directed against PDE5 (1:1,000; Cell Signaling Technology, Inc., #2395S). Anti-GAPDH antibody (1∶1,000; Cell Signaling Technology, Inc., #2118S) was used as a loading control. After incubation for 1 h at RT with a secondary anti-rabbit IgG antibody conjugated to horseradish peroxidase (1∶3,000; Cell Signaling Technology, Inc., #7074S), the proteins were visualized by the WesternSure^®^ PREMIUM Chemiluminescent Substrate (LI-COR Biosciences). Specific bands were quantified with the Odyssey Fc Imaging System (LI-COR Biosciences) (Schmid et al. 2014).

### Cell viability assay

Cells (RD, RH30, and SkMC) were seeded in 96-well plates at a density of 8 × 10^3^ cells per well. After overnight attachment, cells were incubated for 72 h with Sildenafil (Sigma Aldrich; Merck KGaA) in the presence and/or absence of doxorubicin. The cell viability assays were performed in quadruplicates as originally described (Mosmann 1983). Absorbance was measured at 570 nm.

### FACS analysis

Cancer cells were seeded in 6-well plates incubated with the PDE5 inhibitor Sildenafil in the presence and/or absence of doxorubicin for 24 h for ROS-production and 72 h for Apoptosis-Assay. After washing with PBS, cells were subjected to 3.5 µl Annexin V (Biozol) and 3.5 µl Propidium Iodide (PI; Sigma Aldrich; Merck KGaA) or 5 nM 2′,7′dichlorofluorescindiacetate (DCFDA; Sigma Aldrich; Merck KGaA) staining according to the manufacturer recommendations. For ROS measurements, 5 µl 7-AAD (ThermoFisher Scientific) was added to the cell suspension and incubated 15 min at RT in the dark to exclude nonviable from viable cells. Flow-cytometry was performed on BD FACS Canto II flow cytometer (Becton, Dickinson and Company) and evaluated with BD FACS Diva software version 8.0 (Becton, Dickinson and Company).

### Calcium measurements

Fura-2 fluorescence was utilized to determine intracellular Ca^2+^ concentration (Bhavsar et al. 2013). Calcium measurements were performed as previously described (Schmid et al. 2016). In the experiments, RMS cells were pretreated with 20 µM Sildenafil for 24 h.

### Transwell migration assay

To study the migration of tumor cells, transwell migration assays were performed in 24-well plates (Corning, Inc.) using Transwell inserts with an 8-µm pore size (Becton, Dickinson and Company) (Schmid et al. 2012). Cells (RD and RH30) were plated into 6-well plates (Corning Inc.) at 5 × 10^5^ cells per well and pretreated in the presence or absence of Sildenafil (10 and 25 µM). After 24 h, 750 µl of the culture medium with 10% FCS (as a chemoattractant) was pipetted into the lower chambers of 24-well plates. Transwell inserts were placed into each well, and pretreated RH30 and RD cells were transferred into the upper chamber of the inserts at a density of 5 × 10^4^ cells/well in a culture medium without 10% FCS. The cells were either treated or left untreated with Sildenafil for 24 h (RH30) or 48 h (RDs) in a humidified atmosphere of 37 °C and 5% CO_2_. Cells that did not migrate through the pores were removed using a cotton swab and PBS wash. Subsequently, the transwells were moved to 4% paraformaldehyde (Carl Roth GmbH & Co. KG), incubated for 15 min at RT, washed twice with PBS, removed with a scalpel, placed on slides, and stained with Giemsa (Sigma-Aldrich; Merck KGaA). The migrated cells bound on the lower surface of the membrane were then counted with an inverted Axiovert 135 light microscope, at 5 × magnification (Carl Zeiss AG) using three different areas of each membrane. The mean value was calculated using AxioVision Rel 4.8 software (Carl Zeiss AG).

### Colony-forming assay

Colony-forming assays were performed as previously described (Sorg et al. 2021). Number of colonies (> 50 cells) were counted microscopically (Schmid et al. 2016). Dividing the number of colonies by the number of plated cells and multiplying by 100 yielded the colony formation rate according to Franken et al. (Franken et al. 2006).

### Evaluation of drug interaction

To evaluate the effects of the combination of Sildenafil with doxorubicin the coefficient of drug interaction (CDI) was calculated as previously described by Foucquier and Guedj (Foucquier and Guedj 2015). According to the bliss independence model, which differentiates additive from synergistic and antagonistic effects respectively, CDI was calculated by the formula CDI = [(A + B)–(A × B)]/AB, where AB is the ratio of the absorbance in the combination of drugs vs. that of the control, while A or B is the ratio of the absorbance of the single-agent group to that of the control group. CDI values smaller, equal or greater than 1 indicate that the drug act in a synergistic, additive or antagonistic manner, respectively.

### Statistics

Data are provided as means ± standard error (SEM), *n* represents the number of independent experiments. All experiments were repeated at least three times and were tested for significance using unpaired Student’s *t* test with Welch’s correction or one-way ANOVA (Bonferroni or Dunnett correction) using GraphPad Prism version 8.0 (GraphPad Software, Inc.). Results with *p* < 0.05 were considered statistically significant.

## Results

### Expression of PDE5 in RMS cells

**Fig. 1:**
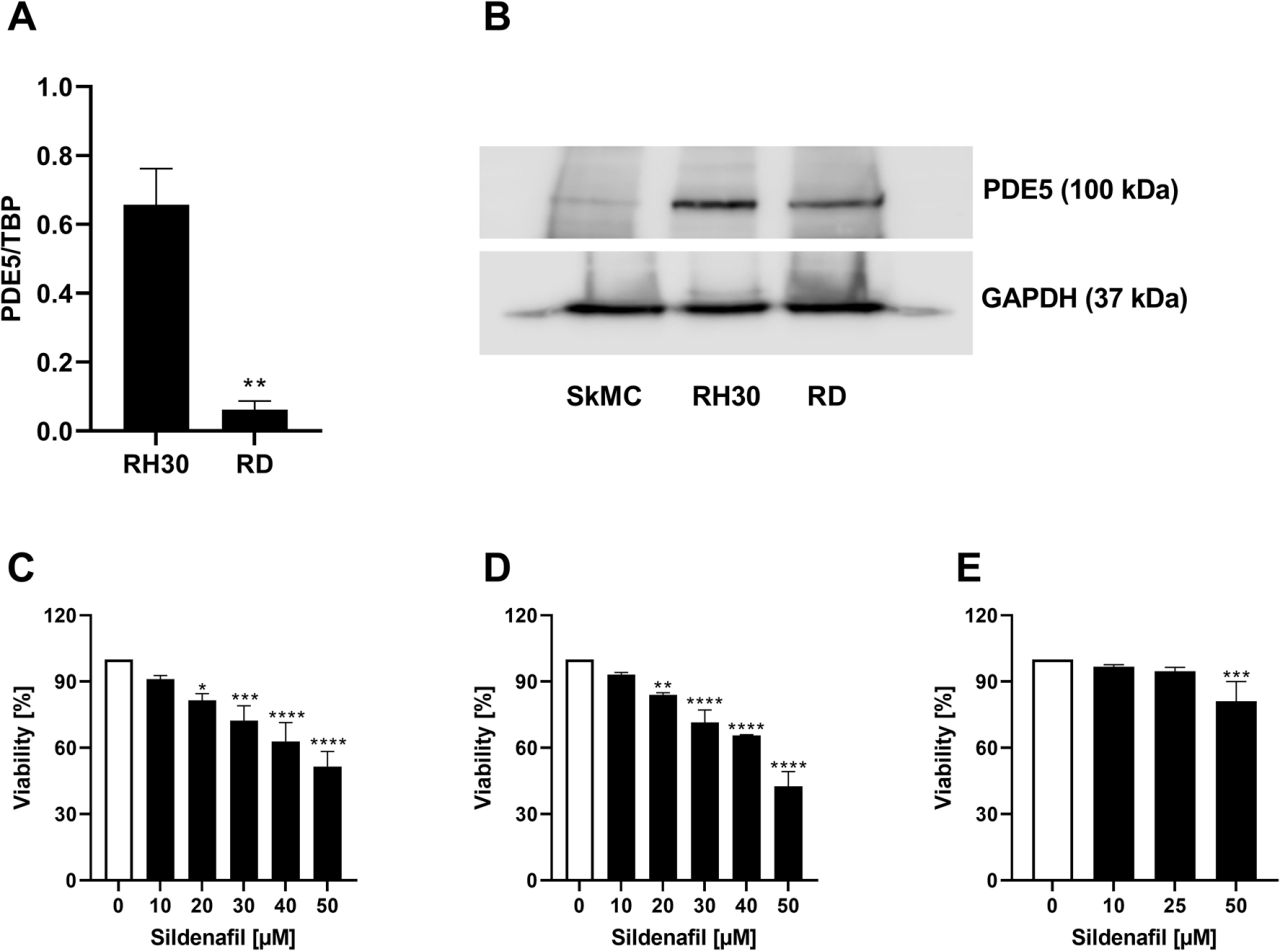
Transcript levels and protein abundance of PDE5 in RD and RH30 cells and the effect of the PDE5 inhibitor sildenafil on the viability of RD and RH30 rhabdomyosarcoma cells as well as SkMCs. Arithmetic means ± SEM (*n* = 4) of PDE5 relative to TBP transcript levels in RD and RH30 cells. **(*p* < 0.01) indicates statistical significance (ANOVA, Student *t* Test with Welch correction) (**A**). Original Western blots showing the protein abundance of PDE5 in RD, RH30 and SkMC cells (**B**). GADPH expression was used as a loading control. Arithmetic means ± SEM (*n* = 3) of the relative numbers of viable RD (**C**), RH30 (**D**) and SKMC (**E**) cells following a 72 h incubation in the presence of sildenafil (black bars) relative to the absence of sildenafil (white bar). *(*p* < 0.05); **(*p* < 0.01); ***(*p* < 0.001), ****(*p* < 0.0001) indicates statistical significance to untreated control (ANOVA, Dunnett correction)

The present study addressed the expression and functional significance of PDE5 in RMS cell lines RD and RH30. PDE5 transcription and protein levels were determined in alveolar (RH30) and embryonal (RD) RMS cell lines. As illustrated in Fig. 1A, PDE5-mRNA expression was significantly higher in alveolar than in embryonal RMS cells. PDE5 protein levels were higher in RH30 cells compared to RD cells and SkMCs (Fig. 1B).

### Effects of Sildenafil on viability of RMS cells

Sildenafil has been previously reported to inhibit the proliferation of various cancer cell lines (Chen et al. 2020). As illustrated in Fig. 1C, D Sildenafil treatment resulted in a significant dose-dependent decline of cell viability in both RMS cells after 72 h. Incubation with 20 µM Sildenafil led to a 19% decrease in RD cells’ viability and a 16% decrease in viability of RH30 cells. The dose of 50 µM Sildenafil led to a viability reduction in RD and RH30 cells of 48% and 58%, respectively. Only treatment with 50 µM Sildenafil resulted in a viability reduction of 20% in SkMC cells (Fig. 1E).

### Effects of Sildenafil on the store-operated calcium entry (SOCE) in RMS cells

**Fig. 2:**
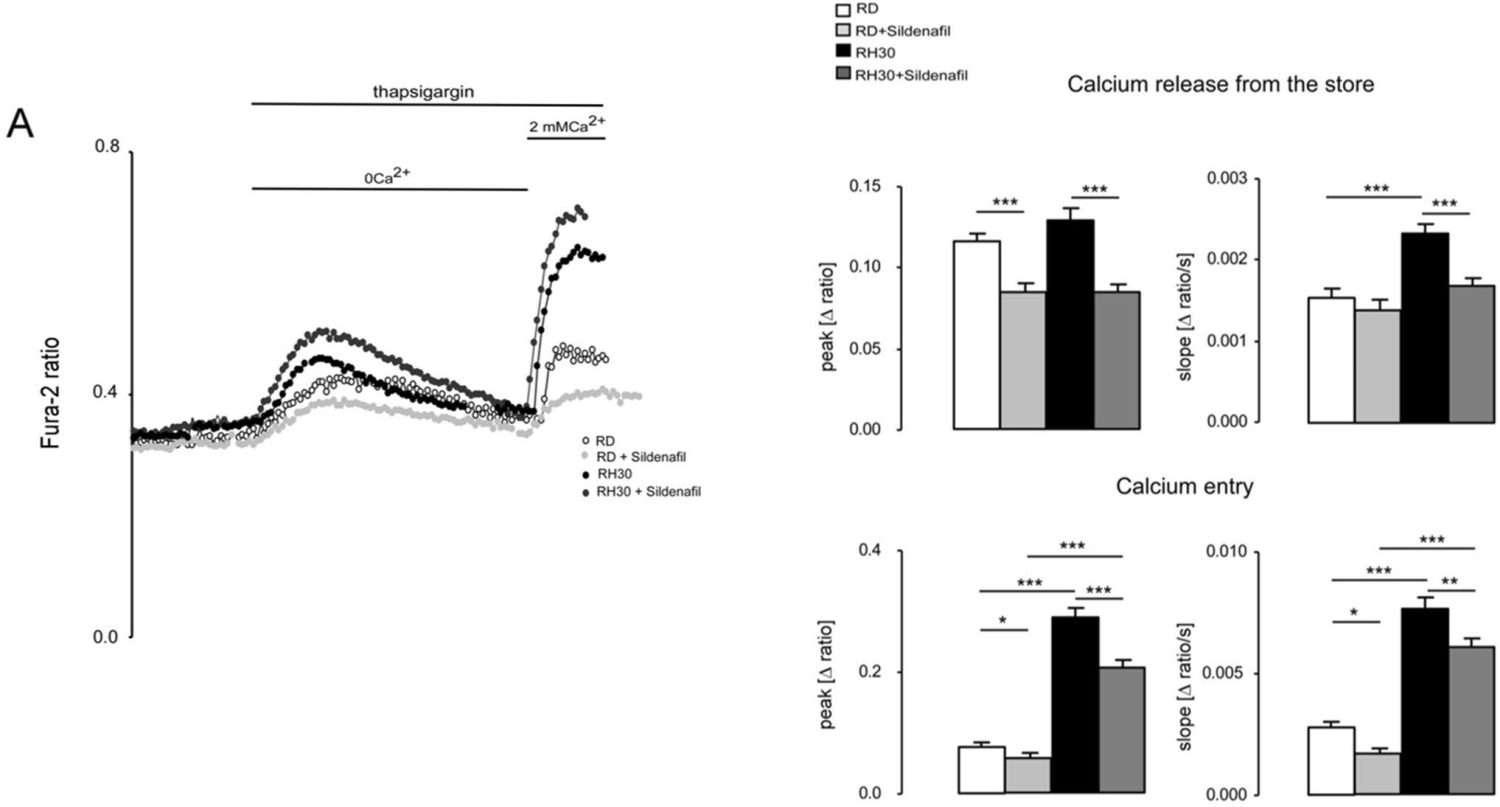
Effect of sildenafil on intracellular Ca^2+^ release and store-operated Ca^2+^ entry (SOCE) in RD and RH30 cells. Representative original tracings of Fura-2 fluorescence-ratio in fluorescence spectrometry prior to, during and after extracellular Ca^2+^ removal and addition of thapsigargin (1 mM), as well as re-addition of extracellular Ca^2+^ in RD (white symbols) and RH30 (black symbols) cells or with a prior 24 h treatment with 20 µM sildenafil in RD (light gray symbols) and RH30 (dark gray symbols) cells (**A**). Arithmetic means ± SEM (*n* = 3) of peak and slope increase of fura-2-fluorescence-ratio from intracellular stores (upper bars) and upon SOCE (lower bars) in RD (white bars) and RH30 (black bars) without (left bars) and with sildenafil (20 µM) treated (right bars) rhabdomyosarcoma cells. *(*p* < 0.05), **(*p* < 0.01), ***(*p* < 0.001) indicates statistically significant difference (ANOVA, Bonferroni correction)

Since both RMS cell lines showed a significant impairment of proliferation at a concentration of 20 µM Sildenafil, calcium measurements were performed using this dosage. Calcium measurements were undertaken to analyze whether Sildenafil has an influence on the intracellular calcium concentration [Ca^2+^]_i_ in RD and RH30 cells. Fura-2 fluorescence of Fura-2-AM loaded RMS cells was taken as a measure of [Ca^2+^]_i_. Store-operated calcium entry (SOCE) was triggered by depleting intracellular calcium stores through extracellular calcium removal and addition of sarcoplasmic reticulum Ca^2+^ ATPase (SERCA) inhibitor thapsigargin followed by subsequent re-addition of extracellular 2 mM Ca^2+^. As illustrated in Fig. 2, levels of calcium concentration (peak) during calcium release from intracellular stores were similar in both cell lines, but the increase in calcium concentration (slope) was significantly faster in RH30 compared to RD cells. Interestingly, the peak and the slope of calcium entry (SOCE) were significantly different between the two tumor cell lines. In the same series of experiments, it was tested, whether [Ca^2+^]_i_ is sensitive to Sildenafil. As shown in Fig. 2, SOCE was significantly higher in RH30 than in RD cells after 24 h pretreatment with 20 µM Sildenafil. However, in both cell lines SOCE was significantly reduced compared to untreated cells.

### Migratory response of RMS cells to Sildenafil

**Fig. 3:**
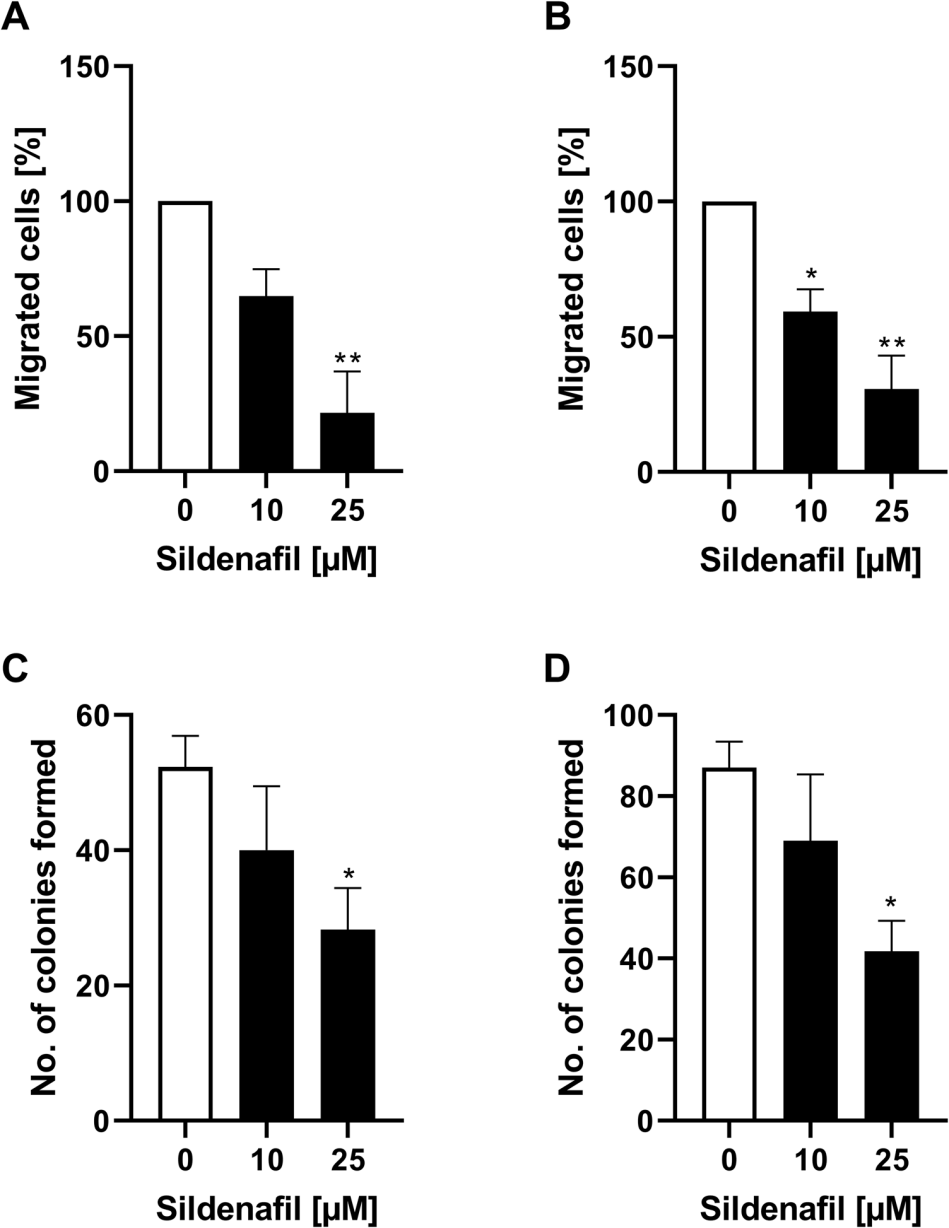
Modulation of sildenafil on migration and colony formation of RMS cells. Arithmetic means ± SEM (*n* = 4) of the percentage migrated RD (**A**) and RH30 cells (**B**) in the absence (white bar) and presence of 10 µM and 25 µM sildenafil (black bars). Arithmetic means ± SEM (*n* = 6) of the relative numbers of evolving clones of RD (**C**) and RH30 (**D**) cell following incubation for 72 h in the absence (white bar) and presence of 10 µM and 25 µM Sildenafil (black bars). *(*p* < 0.05), **(*p* < 0.01) indicates statistically significant difference to untreated control (ANOVA, Dunnett correction)

Given that Sildenafil significantly reduces SOCE in both RD and RH30 cell lines, migration assays were performed to demonstrate the functional effects of this reduction of [Ca^2+^]_i_. As shown in Fig. 3, the percentage of RD and RH30 cells that migrated through a transwell chamber was significantly decreased by about 40% in the presence of Sildenafil at a concentration of 10 µM in RH30 cells and by about 75% at a concentration of 25 µM in RD cells.

### Effects of Sildenafil treatment on colony formation

Not only the migration through the [Ca^2+^]_i_ can be modulated, but among other things, also the ability for clonal growth can be influenced. The impact of Sildenafil on tumor cell proliferation was quantified in colony-forming assays. As presented in Fig. 3, relative numbers of colonies were significantly decreased by about 50% in the presence of 25 µM Sildenafil in both cell lines.

### Effects of Sildenafil in combination with doxorubicin on the viability of RMS cells

**Fig. 4:**
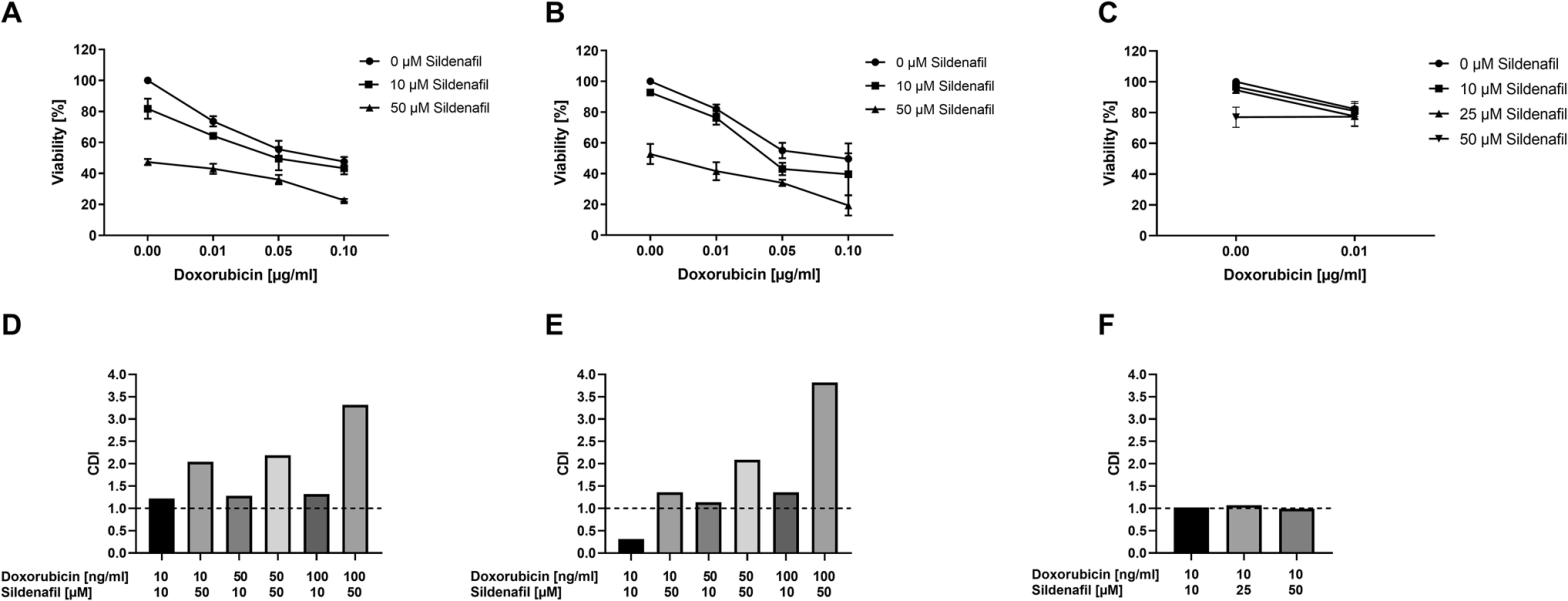
Inhibitory effect of the PDE5 inhibitor sildenafil in the presence or absence of doxorubicin on the viability of RD and RH30 rhabdomyosarcoma cells as well as SkMCs. Arithmetic means ± SEM (*n* = 5) of the relative numbers of viable RD (**A**), RH30 (**B**) and SKMC (**C**) cells following a 72 h incubation with 10 or 50 µM sildenafil in the presence or absence of 0.01, 0.05, and 0.1 µg/ml doxorubicin (10, 50, 100 ng/ml doxorubicin) relative to the absence of sildenafil. *(*p* < 0.05) indicates statistical significance (ANOVA, Bonferroni correction); Calculated coefficient of drug interaction (CDI) for RD (**D**), RH30 (**E**), and SKMC (**F**) cells. CDI values smaller, equal or greater than 1, indicate that the drug act in a synergistic, additive or antagonistic manner, respectively

A further series of experiments should explore whether Sildenafil modifies the effect of doxorubicin concerning cell viability. As shown in Fig. 4, RD and RH30 cells’ viability significantly decreased after treatment with doxorubicin for 72 h. This effect was significantly enhanced by the simultaneous administration of Sildenafil, also in a dose-dependent manner. A synergistic effect was observed in RH30 cells when combining 10 µM Sildenafil with 0.01 µg/ml (10 ng/ml) doxorubicin, compared with the corresponding agents’ effects alone. No synergistic effect was observed in RD cells. Treatment with Sildenafil in the presence of doxorubicin resulted in a slight viability change in SkMCs.

### Migratory response of RMS cells to Sildenafil

**Fig. 5:**
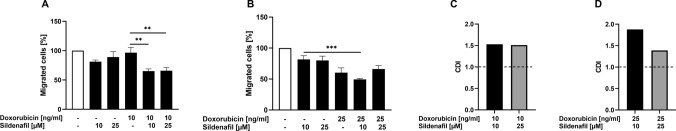
The influence of sildenafil in combination with doxorubicin on the migration of RMS cells. Arithmetic means ± SEM (*n* = 5) of the percentage migrated RD (**A**) and RH30 cells (**B**) in the absence (white bar) and presence of 10 µM and 25 µM sildenafil with and without doxorubicin (black bars). **(*p* < 0.01), ***(*p* < 0.001) indicates statistically significant (ANOVA, Bonferroni correction); Calculated coefficient of drug interaction (CDI) for RD (**C**), RH30 (**D**) cells. CDI values smaller, equal or greater than 1, indicate that the drug act in a synergistic, additive or antagonistic manner, respectively

Given that Sildenafil significantly reduces migration in both RD and RH30 cell lines, migration assays were performed to demonstrate that Sildenafil modifies the effect of doxorubicin concerning migration. As shown in Fig. 5A, the percentage of RD cells that migrated through a transwell chamber was significantly decreased by about 40% in the presence of Sildenafil and doxorubicin in comparison with doxorubicin alone. In RH30 cells the migration was significantly decreased by about 60% when cells were incubated with 10 µM Sildenafil and doxorubicin in comparison with 10 µM Sildenafil alone (Fig. 5B). No synergistic or additive effect was observed in RD (Fig. 5C) and RH30 (Fig. 5D) cell lines.

### Effects of Sildenafil in combination with doxorubicin on the proportion of apoptotic cells and production of ROS in RMS cells

**Fig. 6:**
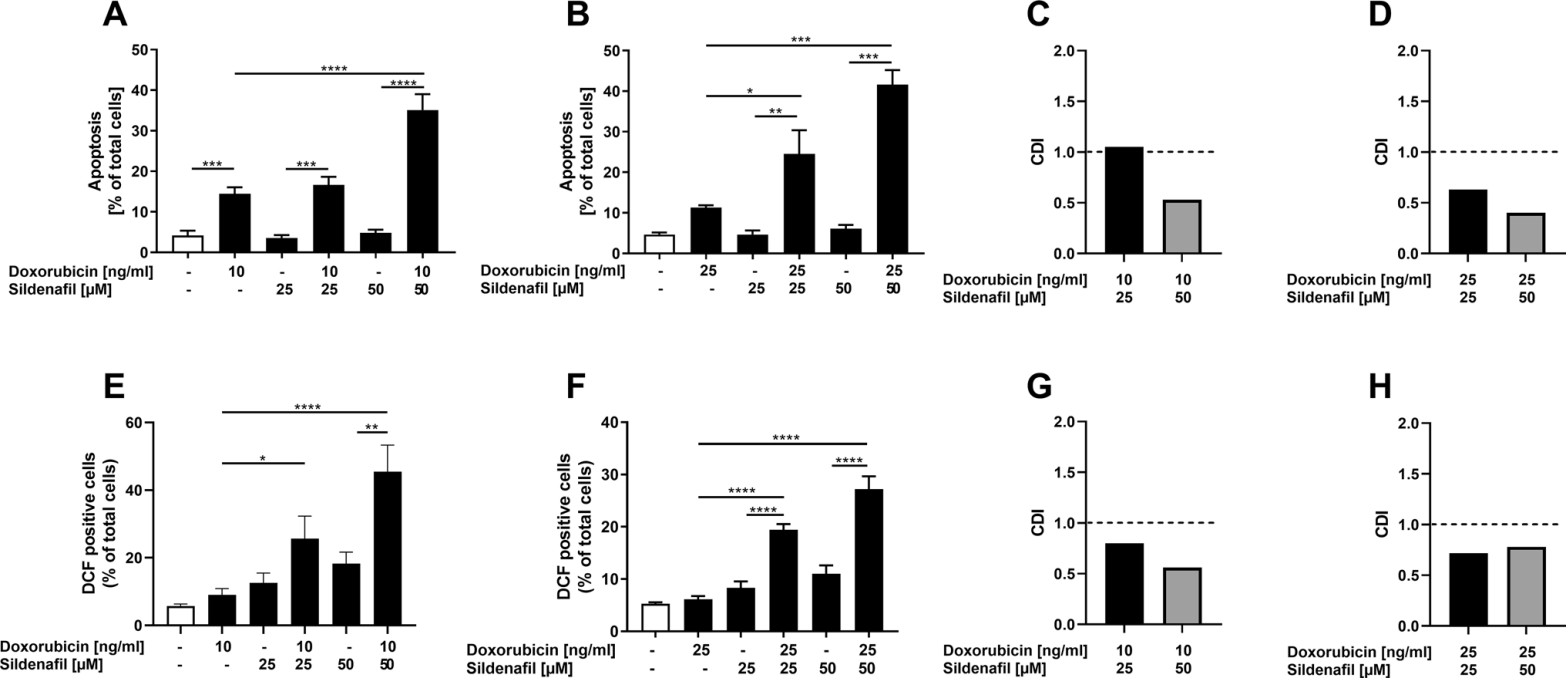
Modulation of sildenafil in combination with doxorubicin on apoptosis and ROS production in RMS cells. Apoptosis: RD (**A**) and RH30 (**B**) cells were treated with 25 µM or 50 µM Sildenafil in the presence or absence of doxorubicin (RD, 10 ng/ml; RH30, 25 ng/ml) for 72 h. Cells were subjected to Annexin V and Propidium Iodide (PI) staining and subsequent flow cytometry. Data are shown as arithmetic means ± SEM (*n* = 4). *(*p* < 0.05); **(*p* < 0.01); ***(*p* < 0.001), ****(*p* < 0.0001) indicates statistical significance (ANOVA, Bonferroni correction); ROS production: RD (**E**) and RH30 (**F**) cells were treated with 25 µM or 50 µM Sildenafil in the presence or absence of doxorubicin (RD, 10 ng/ml; RH30, 25 ng/ml) for 24 h. Cells were treated with DCFDA and measured via flow cytometry. Data are shown as arithmetic means ± SEM (*n* = 4). *(*p* < 0.05); **(*p* < 0.01); ****(*p* < 0.0001) indicates statistical significance (ANOVA, Bonferroni correction); Calculated coefficient of drug interaction (CDI) for RD (**C**, **G**), RH30 (**D**, **H**) cells. CDI values smaller, equal or greater than 1, indicate that the drug act in a synergistic, additive or antagonistic manner, respectively

To investigate whether PDE5 inhibition by Sildenafil in the presence of doxorubicin affected apoptosis in RMS, tumor cells were incubated for 72 h before being stained with Annexin V and PI and subsequently subjected to FACS analysis. As shown in Fig. 6, the proportion of apoptotic cells in both cell lines was significantly higher after incubation with Sildenafil in the presence of doxorubicin compared to incubation with Sildenafil or doxorubicin alone. In RD cells, synergistic effects were observed only when combining 0.01 µg/ml (10 ng/ml) doxorubicin with 50 µM Sildenafil (Fig. 6C). In RH30 cells, all combinations were synergistic (Fig. 6D). To substantiate these findings, FACS analyses evaluating the ROS production were performed. Incubation for 24 h with Sildenafil in the presence of doxorubicin resulted in a substantial increase of ROS production in both cell lines in comparison to incubation with Sildenafil or doxorubicin alone (Fig. 6E, F). In both cell lines, the effects were synergistic for all combinations of the concentrations used (Fig. 6G, H).

## Discussion

The present study investigated the effects of Sildenafil alone and combined with doxorubicin on pediatric RMS cell lines. We found that Sildenafil in combination with doxorubicin significantly reduced cell viability and determined a significant increase in apoptosis rate and ROS production in RMS cells compared to doxorubicin alone. To our knowledge, this is the first report addressing the effect of Sildenafil in combination with doxorubicin in RMS cells in vitro.

DNA-damaging agents have a long history of use in cancer chemotherapy. Doxorubicin is a very potent cytotoxic drug and is commonly used to treat a great variety of cancers, including many types of solid tumors and soft tissue sarcomas (Chang et al. 2018; Weigel et al. 2016; Green et al. 2001). Several studies have demonstrated its efficacy in pediatric patients with high-risk and metastatic RMS (Bonadonna et al. 1970; Bisogno et al. 2018; Bergeron et al. 2008; Sandler et al. 2001). A phase II window study of single-agent doxorubicin in children with high-risk metastatic RMS showed a 65% overall response rate after two courses of 60 mg/m^2^ given at 21 days intervals (Bergeron et al. 2008). A previous phase II window study of combined doxorubicin/ifosfamide in children with newly diagnosed metastatic RMS reported a similar response rate (63%) (Sandler et al. 2001). In children with Wilms tumor, doxorubicin was added to vincristine and dactinomycin for those with stages III and IV favorable histology and significantly improved the relapse free and overall survival rates for children with stage III favorable histology tumors (Green et al. 2001).

Important drawbacks of doxorubicin use are major side effects such as cardiotoxicity and myelosuppression (Wu et al. 2016; Gilladoga et al. 1976). Risk factors for cardiotoxicity include young age, high cumulative dose, and radiation fields, including the heart (Arndt 2012). As RMS’s peak incidence is seen early in childhood (median age at diagnosis: 5 years of age), cardiotoxicity is an important factor in the risk/benefit calculation (Bisogno et al. 2018; Arndt 2012). Additionally, resistance to chemotherapeutic agents is a common event, making administration of higher doses necessary, increasing the drug-induced toxicity (Wu et al. 2016).

Sildenafil is a potent and selective inhibitor of PDE5, and it has been extensively used off-label for the treatment of pediatric pulmonary hypertension since 2005 (Cohen et al. 2019). Sildenafil has been found to enhance different chemotherapeutic agents’ cytotoxic effect including cisplatin, gemcitabine, and doxorubicin (Wille et al. 2014; Das et al. 2016). Das et al. reported that the combination of Sildenafil and doxorubicin enhances the destruction of ovarian cancer and sarcoma cells, suggesting a potential utility of Sildenafil in chemo-sensitization of various malignancies (Das et al. 2010). The same author also observed that the co-treatment of prostate cancer cells with Sildenafil and doxorubicin has led to a reduced expression of FLIP (anti-apoptotic molecule) compared to individual drug treatment (Das et al. 2016).

Therefore, the combination therapy of doxorubicin with non-chemotherapeutic drugs such as Sildenafil might reduce the possible side effects in children by dose reduction while maintaining synergistic anticancer activity in pediatric RMS (Chang et al. 2018).

Mechanisms participating in the orchestration of tumor cell migration, proliferation, and death include timely alteration of cytosolic calcium activity (Schmid et al. 2016). It has been demonstrated that calcium oscillations are involved in stimulating different cellular functions, such as entering into the S and the M phase of the cell cycle (Taylor et al. 2008), and the support of cell survival (Heise et al. 2010). In contrast, a sustained increase of cytosolic calcium promotes the induction of apoptosis (Lang and Hoffmann 2012). There is also accumulating evidence that SOCE plays a key role in regulating cell migration (Jardin and Rosado 2016). Recently we reported for the first time the influence of Orai1 and STIM1 in SOCE in RMS cell lines and found that SOCE inhibitor BTP2 and Orai1 inhibitor 2-APB virtually abrogated SOCE in RMS cells (Schmid et al. 2016). These results suggest that Orai1 signaling is involved in SOCE into RMS cells, contributing to migration, invasion, and proliferation (Schmid et al. 2016).

In the present study, we observed that SOCE in the presence of Sildenafil was significantly reduced in both cell lines compared to untreated cells, suggesting a possible link between PDE5 inhibition and decreased calcium signaling, leading to decreased cell viability, migration, and colony formation.

Most cytostatic drugs kill their target cell at least partly through the generation of large amounts of intracellular ROS (Laurent et al. 2005). ROS can stimulate pro-apoptotic signal molecules, activate the p53 protein pathway or the mitochondrial apoptotic cascade (Laurent et al. 2005; Benhar et al. 2001; Tobiume et al. 2001). Sildenafil has been reported to induce ROS accumulation in cancer cells. Mei et al. demonstrated that the exposure of human colorectal cancer cells to Sildenafil resulted in increased intracellular ROS levels (Mei et al. 2015). The authors also observed that pretreatment of the cells with the ROS scavenger N-acetyl-L-cysteine could reverse the Sildenafil-induced ROS accumulation and cell apoptosis, suggesting that ROS is critical for Sildenafil-induced apoptosis in colorectal cancer cells (Mei et al. 2015). Our study showed that incubation of RMS cells with Sildenafil in the presence of doxorubicin resulted in a substantial increase in ROS levels compared to incubation of RMS cells with either agent alone. Similarly, the proportion of apoptotic cells was significantly higher after combined treatment than after treatment with the corresponding agents alone. Additionally, a synergistic effect of the combination therapy could be observed in both cell lines, suggesting that Sildenafil may potentiate doxorubicin effects.

In conclusion, our results indicate a link between PDE5 inhibition by Sildenafil and decreased calcium signalling, leading to impaired viability, migration, and colony forming in RMS cells. Additionally, the combined treatment potentiated ROS production and apoptosis. Therefore, the combination of Sildenafil with doxorubicin may be a promising approach in the treatment of paediatric RMS. Further studies, including in vivo experiments, should be conducted to assess the possible benefit of affected patients.
